# Granular Conjunctiva

**Published:** 1857-01

**Authors:** C. S. Fenner

**Affiliations:** Memphis, Tennessee


### Original Communications.

### ART. I.—*Granular Conjunctiva.* By C. S. FENNER, M.D., Memphis, Tennessee.

THAT condition of the lining membrane of the upper eyelid, known by the name of granular conjunctiva, the result of purulent ophthalmia, does not seem to have attracted as much attention from European surgeons, as they have devoted to other ocular affections, not more destructive to the eye, and of less frequent occurrence. Mr. Cooper, in his Surgical Dictionary, devotes but little more than half a page to this subject. Mr. McKenzie disposes of it in less than four pages, while he gives twenty-eight to iritis, and ninety-five to cataract. In the Southwest, we have a score of cases of granular lids to treat, to one of iritis or cataract; indeed, more than half of all the diseases of the eye, coming under the notice of the surgeon, is of this character, and it is the cause of a majority of the cases of impaired vision, and entire blindness. Dr. Hays, in his edition of "Lawrence on the Eye," has given by far the best account of the pathology and treatment of this disease that I have seen. In regard to its frequent occurrence, and the want of knowledge of the profession in respect to its proper treatment, he says, "A very large proportion of the applicants for admission into Wills Hospital, labor under this disease; and the treatment which they represent themselves to have undergone, as well as the statements of private patients, leads us to infer that physicians generally are not as well acquainted with this complaint as it is desirable they should be." I propose, in this paper, to give a brief description of granular palpebral conjunctiva, as it occurs in the Southwest, and the plan of treatment I have found, after considerable experience, best adapted to give relief, and produce a final and an effectual cure. I am satisfied that this result cannot be obtained, in many instances, by any of the plans of treatment usually recommended by authors. Mr. Lawrence says, "The palpebral lining becomes thoroughly altered in structure, and we cannot be surprised that it should be difficult, and, indeed, hardly possible, to restore its healthy state." Mr. McKenzie says, "Frequent relapses may, at last, render this disease incurable." Dr. Hays remarks, it "is a most troublesome affection,—extremely obstinate,—subject to sudden and violent exacerbations from the slightest causes, and therefore demanding the incessant attention of the practitioner; and when allowed to continue, tending surely to the impairment, and most generally, indeed, the total destruction of vision." Every surgeon who has seen much of this disease, and attempted to treat it, will agree with the opinions expressed by the abovenamed authors, that the restoration of granular palpebral conjunctiva to a healthy state, is, at all times, difficult to accomplish, and requires much perseverance on the part of the professional attendant. It is not my purpose to speak of acute purulent ophthalmia, previous to this diseased condition of the lids, but I would remark, that I believe that if the original acute inflammation were promptly and judiciously treated at its outset, the granular state would seldom occur. The disease is the result of a long-continued high state of inflammation, which has been permitted to run its course, either from neglect or injudicious treatment. Very acute purulent ophthalmia, if checked early, will usually terminate in a natural condition of the lid.

We find granular conjunctiva presenting several different varieties.

1st. The everted lid has a villous appearance, the natural papillæ of the membrane are elongated, without much enlargement; they are very red, and evenly spread over the whole mucous surface. This form of the disease is attended with considerable redness of the ocular conjunctiva, lachrymation, and increased sensibility to light, but is not liable to frequent recurrences of acute inflammation.

2d. The inner surface of the lid appears as if spread over with bruised muscular fibre, is highly engorged with blood, and bleeds on the slightest touch. The fold of conjunctiva extending from the lower lid to the eye is much swollen, and engorged with dark venous blood, and, if the lid be depressed, rises up so as to touch or overlap the edge of the cornea. The caruncula lachrymalis is also swollen; and there is enlargement of the Meibomian follicles, with increased secretion. This form of the disease is subject to frequent and violent exacerbations, beginning with a stinging pain, usually at the external canthus, increased redness of the eye, swelling of the lids, severe supra-orbital pain, and other symptoms of acute conjunctivitis.

3d. The conjunctiva is converted into wart-like excrescences of various sizes, with deep sulci running between them.

4th. The lining membrane of the lid is thickened, and presents something of a cartilaginous appearance. The membrane is contracted, rendering it difficult to evert the lid, and when turned, the blood is forced out, so that the part appears white and glistening. The lids do not open as wide as usual, causing the eye to appear smaller than natural. This condition is more frequently found in middle and advanced age, and is attended with considerable opacity of the cornea and dimness of vision, but is less liable to relapses than the second and third varieties.

Granulations of the conjunctiva are spoken of by modern authors as a hypertrophy of the natural papillæ of the mucous membrane. In the first variety I have described, this is undoubtedly correct, but in the other forms there seems to be a complete change of structure. The cornea, at first, is not usually seriously affected, but the rough, diseased lid, constantly rubbing over its surface, causes thickening and opacity of its superficial layer, and enlargement of its arteries, which may be seen running from above downwards in considerable numbers; and if there is a small superficial ulcer, a large artery is generally found running from the temporal or nasal side of the cornea, and terminating at the ulcer. Upon every exposure, or atmospheric change, and indeed, frequently without any perceptible cause, acute inflammation comes on, attended with swelling of the lids, increased purulent discharge, photophobia, pain in the forehead, temple, cheek, and often soreness over the entire scalp. The vascularity and opacity of the cornea increase, and are not unfrequently followed by onyx, hypopyon, and extensive ulceration, terminating in synechia anterior, albugo, staphyloma, &c. More generally, the acute symptoms subside before the latter disorganizing results occur; the cornea slowly clears up, and the patient flatters himself that he will soon be well, but another relapse occurs, and his hopes are disappointed.

Each exacerbation adds to the injury of the cornea, until, after a longer or shorter period, varying in different persons, changes take place in the eye, as above mentioned, beyond the power of nature and art to remedy, and the patient becomes incurably blind. This is the usual course of the disease for the second and third varieties, but the first and fourth often remain for a long time without much change. In the first variety described, there are considerable redness, sensibility to light, and vascularity of the upper edge of the cornea, without much opacity. In the fourth or cartilaginous form, there is less vascularity of the cornea, but considerable opacity, and often superficial ulceration, extending barely through the conjunctival coat. Granular conjunctiva is at all times attended with an increased secretion of mucus, in the morning glueing the eyelids together, and during the day spreading over the cornea, and to some degree obstructing vision.

*Treatment.*—This may be divided into constitutional and local. The first important requisite in treating diseased palpebral conjunctiva is to obtain the patient's entire confidence. It is well, after examining him, and before asking any questions, to give a pretty correct history of his case, particularly in reference to the original purulent attack, his improvement and hope of speedy recovery, followed by exacerbation after exacerbation, until he has nearly despaired of ever being restored. A tolerably accurate description, thus given, will inspire the patient with confidence in regard to the professional attendant's knowledge of his case, and will make him listen to advice, and more patiently submit to the prolonged treatment requisite to restore his lids to a healthy condition. The nature of his case should be fully explained to him, and the difficulties attending its cure; that it will require months of unremitting attention; that in all probability he will have occasional relapses, even after he regards himself as almost well; that he must implicitly follow the directions given him in reference to treatment, exposure, &c. Promising the patient to cure a bad case of granular lids in a short time may be very gratifying to him; but if that end fails to be accomplished in the specified period, he becomes desponding, dissatisfied with the treatment, loses confidence in his physician, and seeks another professional adviser.

I will first speak of general or constitutional treatment. Those who have suffered long from this complaint generally represent themselves to have been under the treatment of several physicians, most of whom promised a speedy cure; to have been blistered, bled, purged, starved, kept for weeks at a time in a dark room, "eye-waters" of various kinds dropped into the eyes, and all without any permanent benefit; the disease constantly getting worse. My practice is to put the patient upon a generous diet, and endeavor to raise his system and strength to a full standard of health; and direct him to expose his eyes to as much light as he can bear without pain, and allow the atmospheric air to come in contact with them. If, however, he becomes too plethoric, or a relapse occurs, threatening the safety of the cornea, blood should be drawn from the arm. The great object to accomplish, without which all local treatment for the restoration of the diseased conjunctiva will be unavailing, is to prevent the recurrence of attacks of acute inflammation, one of the characteristics of the disease, and which, in the changeable climate of the Southwest, is of very frequent occurrence, particularly in the winter and spring months. I have tried nearly every plan of treatment recommended by authors to accomplish this object, and generally without success. I have used quinine, arsenic, hydriodate of potash, veratria, wine of colchicum, mercury, opium, blisters, setons, &c., without any permanent benefit. Quinine, in ten-grain doses, will generally cut short the exacerbation, and relieve the supraorbital pain, but does not prevent a recurrence. I have noticed that relapses usually take place in numbers of cases of this disease simultaneously. For instance, if I have a dozen patients under treatment, and all are improving, they all or nearly all have a relapse about the same time, although most of them assure me they have not been imprudent, or exposed themselves in any way. I have sometimes been able to predict that my patient would be worse in the morning, from feeling a rheumatic pain in my own shoulder the preceding night, and generally my prediction has proved correct. Indeed, of so little avail have I found ordinary treatment, in preventing exacerbations, that for several years I was in the habit of assuring those who came to me with this disease in the winter or spring, that I knew of no means of preventing their occasionally getting worse during the atmospheric changes of the season; that the object of treatment would be to prevent those attacks as much as possible, and in the meanwhile I would apply local treatment to the lid until steady warm weather should set in, beginning about the middle of May, when, in all probability, the relapses would cease for several months, and during that time the lid could be brought to such a condition, and the eye regain its powers, so as to be able to resist the effects of subsequent atmospheric influences. Patients usually get much better even without any treatment during the summer months, and entertain high hopes of recovery, but without appropriate local treatment, a relapse takes place in the fall.

Regarding these exacerbations, accompanied with circumorbital pain, soreness in the periosteum and scalp, as of rheumatic origin, about two years ago I was induced to give a trial to the phytolacca decandra or poke, from its well-known efficacy in relieving rheumatic affections, and the result has far exceeded my most sanguine expectations. With the aid of this remedy, I have been enabled to effectually cure cases of granular conjunctiva, that, without it, would have resisted all my efforts; indeed, with me it has proved almost a specific for the exacerbations attending this complaint. Patients fully under the influence of the phytolacca, often expose themselves and take a severe cold without affecting the eyes in the least. I make use of the root, and prescribe it either in the form of a very strong decoction, or tincture; the former I prefer, as less liable to nauseate or act on the bowels. I direct a half peck of the root cut in small pieces, to be put into a kettle, to which is added four quarts of water, to be boiled down to one quart and strained. Of this a wineglass full may be taken every two or three hours. Some patients require more than others. The dose should be sufficient to produce a fulness of the temples and head a few minutes after it is taken, and patients soon learn to know the quantity required to produce this effect. Besides the fulness of the head, it causes flushing of the face, a general glow and perspiration over the entire surface of the body, often fulness of the stomach, and occasionally nausea. After having been used four or five days, it usually acts on the bowels, when an opiate should be administered as occasion may require, and the quantity of the decoction diminished for a time, to be increased, however, on every unfavorable change of the weather, or the slightest symptom of a relapse. I have not yet seen a severe recurrence of acute inflammation in this disease, where the patient was kept fully under the influence of the phytolacca. If there is ulceration of the cornea, or much opacity, I usually prescribe a pill composed of one grain of calomel and the fourth of a grain of opium, to be taken every night. I know of no remedy so efficacious in promoting absorption of lymph deposited in the texture of the cornea as mercury, either in the form of calomel or blue mass, or, if these remedies are found to act on the salivary glands, I use the corrosive chloride, combined with the compound syrup of sarsaparilla. The latter form of mercury rarely salivates; it may be continued for months, and is particularly adapted to strumous cases attended with severe photophobia. If the system has been much reduced, and is in an anæmic condition, the preparations of iron will be of service.

*Local Treatment.*—There has been, and is yet, a great variety of opinion among surgeons in regard to the proper local treatment of granular conjunctiva, and a great many stimulating, caustic, and astringent applications, have been recommended, most of which I have found absolutely injurious. The only articles I now use locally are a saturated solution of the acetate of lead, the undiluted liquor plumbi diacetatis, the sulphate of copper, and occasionally the knife. In the fourth variety I have described, where the lining membrane is contracted, white when everted, and having the appearance of cartilage, I have found the saturated solution of the acetate of lead brushed over the diseased surface every morning restore the parts in a very few weeks. The solution should be applied for two or three minutes, with a camel's hair pencil. This remedy answers better than any other in simple swelling of the conjunctiva remaining after the first attack of purulent ophthalmia, when the inflammation of the eye has subsided, and before repeated exacerbations have changed the structure of the membrane. In the other varieties, I have found it of service alternated with sulphate of copper. When the granules are loose and spongy, a few applications of the acetate of lead will contract and harden them, after which its efficacy seems to cease, when the sulphate of copper will be more beneficial. The lead should be applied until the part becomes of a milky whiteness, caused by the coagulation of the albumen of the blood, after which a stream of warm water from a sponge should be passed over the lid, before it is permitted to fall back to its place. If, on the next day, the whiteness has nearly disappeared, the same application should be repeated. Sometimes the whiteness remains for several days without any perceptible change, when the remedy will generally be found to irritate the eye, and not adapted to the case. I have seen the lead continued until there seemed to be a white deposit on the diseased surface, beneath which the parts were extremely sensitive, and inclined to bleed upon the slightest irritation.

In the first three varieties of the disease, more benefit will result from the local application of the sulphate of copper than from any other remedy; and I believe that this article, occasionally alternated with the solution of the acetate of lead, will accomplish all that can be desired. A firm, well-crystallized piece of the salt should be selected, cut to a wedge-shape, and fastened in a quill. The lid should be everted, and the mucus wiped away with a soft sponge; the end of the copper should be dipped in water, and rubbed over the entire diseased surface until it becomes of a dirty white or greenish color, when a stream of water should be allowed to flow over the surface, before the lid resumes its natural position. This is to be repeated every morning. It causes considerable pain and redness of the eye, which soon passes away, and the relief is so marked that the patient soon learns to prefer this application to any other. Under this treatment the spongy granulations contract, harden, become flattened on the surface, the fissures gradually disappear, until the whole surface is nearly smooth, although the membrane remains thickened. At this stage patients are so much relieved that they often consider themselves well, and dislike to submit to further treatment, particularly if they are at a distance from home, or think they can continue the applications with the assistance of a friend. The first point of healthy conjunctiva is seen at the edge of the tarsus, in the centre of the lid; a few bloodvessels will be discovered running from the edge, and lost in the diseased membrane. These vessels become shorter on either side of the centre of the lid, until they disappear. Gradually the arteries extend, until the whole surface assumes a healthy appearance, the temporal and nasal portions being the last to yield. The healthy conjunctiva seems to extend from the edge of the tarsus, in the same manner that the skin spreads over a denuded surface.

I was formerly in the habit of using the knife freely in the beginning of the treatment, shaving off the fleshy growth, but I found no benefit from that course—a diseased surface still remaining, from which the granulations sprung as freely as before—and for a long time I entirely abandoned its use; but I have subsequently found it beneficial, after the sulphate of copper has been used until the granulations are hardened and contracted; then, shaving off the surface of some of the most prominent near the edge of the tarsus, and immediately applying the copper, I have found to hasten the cure. Nitrate of silver, either in solution or substance, which has been so much lauded as an application to granular conjunctiva, has in my hands proved injurious, causing swelling of the lids, inflammation, and not unfrequently ulceration of the cornea. It does not produce the kind of action necessary, which should be actively stimulating and astringent, to produce contraction and absorption of the granules, rather than their destruction by caustic substances. During the exacerbations I have found the solution of the nitrate of silver, of the strength of from twenty to thirty grains to the ounce of water, more efficacious than any other remedy, when applied with a brush to the engorged fold of conjunctiva extending from the lower lid to the eye. I have tried the iodide of zinc, as recommended by Dr. Hays, but the result has not proved satisfactory. A solution of common salt is sometimes of service in mild cases, and makes a very good occasional wash for the eye during the day, to remove any slight roughness or smarting, and to clear the cornea of a thin stratum of mucus, that accumulates on its surface. Active escharotics, according to my experience, should never be employed; they leave an abnormal surface, from which the disease is rapidly reproduced. They prevent the parts from ever assuming a healthy state, and the inflammatory action they induce rarely fails to injure the eye. Mr. Lawrence remarks: "After the use of escharotics, the conjunctiva does not regain its normal state; it exhibits traces of the former affection, which, however, do not interfere with its function. It is thicker and has a leathery appearance, with a darker red color than in the natural state, and sometimes we observe whitish cicatrices." Walther's remarks in reference to the escharotic plan of treatment are highly appropriate. He says: "The benefit derived from them is, on the whole, inconsiderable; even when methodically and cautiously employed, they either do not effect a complete cure, or bring it about very slowly. . . . Most of them are so strong, that the eye, even in its relaxed state, will not bear them without experiencing inflammatory reaction. . . . I am indeed astonished when I see one of the most delicate organs attacked with a series of applications so powerful and destructive, from corrosive sublimate to arsenic. The number of these local remedies is calculated to excite distrust. When a disease can be easily and safely cured, the remedies are few, simple, and recommended by reason and experience. They become multiplied in proportion to the obstinacy and tediousness of the complaint." Counter-irritation by means of a seton or blister to the back of the neck, will be found serviceable, and the frequent bathing of the eye during the day in warm water, will give much relief. The above plan of treatment regularly and systematically pursued, will rarely fail to bring about a perfectly healthy condition of the palpebral conjunctiva, and a complete restoration of vision, where the eye has not been injured beyond the power of reparation.

